# A Strong Evidence Outbreak of *Salmonella* Enteritidis in Central Italy Linked to the Consumption of Contaminated Raw Sheep Milk Cheese

**DOI:** 10.3390/microorganisms9122464

**Published:** 2021-11-29

**Authors:** Maira Napoleoni, Laura Villa, Lisa Barco, Luca Busani, Veronica Cibin, Claudia Lucarelli, Alessia Tiengo, Anna Maria Dionisi, Fabrizio Conti, Fernanda Rogeria Da Silva Nunes, Luana Tantucci, Monica Staffolani, Valentina Silenzi, Roberta Fraticelli, Benedetto Morandi, Giuliana Blasi, Elena Rocchegiani, Stefano Fisichella, on behalf of the Enter-Net and Enter-Vet Peripheral Laboratories Referents for Marche Region

**Affiliations:** 1 Istituto Zooprofilattico Sperimentale dell’Umbria e delle Marche “Togo Rosati”, Centro di Riferimento Regionale Patogeni Enterici e Laboratorio Controllo Alimenti, Via Maestri del Lavoro, 7, 62029 Tolentino, Macerata, Italy; m.staffolani@izsum.it (M.S.); v.silenzi@izsum.it (V.S.); g.blasi@izsum.it (G.B.); e.rocchegiani@izsum.it (E.R.); s.fisichella@izsum.it (S.F.); 2 Istituto Superiore di Sanità, Dipartimento di Malattie Infettive, Viale Regina Elena, 299, 00161 Roma, Italy; laura.villa@iss.it (L.V.); claudia.lucarelli@iss.it (C.L.); annamaria.dionisi@iss.it (A.M.D.); 3 Istituto Zooprofilattico Sperimentale Delle Venezie, Centro di Referenza Nazionale e Laboratorio di Referenza OIE per le Salmonellosi, Viale dell’Università, 10, 35020 Legnaro, Padova, Italy; lbarco@izsvenezie.it (L.B.); vcibin@izsvenezie.it (V.C.); atiengo@izsvenezie.it (A.T.); 4 Istituto Superiore di Sanità, Centro di Riferimento per la Medicina di Genere, Reparto Prevenzione e Salute di Genere, Viale Regina Elena, 299, 00161 Roma, Italy; luca.busani@iss.it; 5 Azienda Sanitaria Unica Regionale Marche, Area Vasta 2, Dipartimento di Prevenzione, Via Guerri, 9/11, 60035 Jesi, Ancona, Italy; fabrizio.conti@sanita.marche.it (F.C.); fernanda.dasilva@sanita.marche.it (F.R.D.S.N.); luana.tantucci@sanita.marche.it (L.T.); 6 Istituto Zooprofilattico Sperimentale dell’Umbria e delle Marche “Togo Rosati”, Laboratorio Diagnostica Animale, Via Maestri del Lavoro, 7, 62029 Tolentino, Macerata, Italy; r.fraticelli@izsum.it (R.F.); b.morandi@izsum.it (B.M.)

**Keywords:** *Salmonella* Enteritidis, raw milk, pecorino “primo sale” cheese, sheep, udder colonization, foodborne outbreak

## Abstract

Salmonellosis is the second most commonly reported gastrointestinal infection in humans after campylobacteriosis, and an important cause of foodborne outbreaks in the EU/EEA. The vast majority (72.4%) of the salmonellosis foodborne outbreaks reported in EU in 2019 were caused by *Salmonella* Enteritidis, even if their total number due to this serovar decreased. In spring 2020, a foodborne outbreak of *S.* Enteritidis occurred in the Marche region (Central Italy), involving 85 people. The common exposure source was a cheese, pecorino “primo sale”, produced with raw sheep milk. The cheese batches were produced by two local dairies, with a livestock production facility, also including a sheep farm, being part of one dairy. Bacteriological analysis of samples collected allowed the detection of *S.* Enteritidis in animal faeces, environmental samples, raw-milk bulk tanks and milk taken from single animals. These data confirm that, despite the scarce scientific evidence, *S.* Enteritidis can infect sheep and be shed into the animals’ milk. Hence, this is a real risk for public health when unpasteurized milk is used in production of such cheese. The present paper describes the results of the investigations conducted to clarify this outbreak.

## 1. Introduction

*S.* Enteritidis one of the most important *Salmonella* serovars transmitted from animals to humans worldwide and the most common serovar isolated from human cases in Europe. In 2019, 87,923 confirmed cases of salmonellosis in humans were reported with an EU notification rate of 20.0 cases per 100,000 population, which was at the same level as in 2018. The trend for salmonellosis in humans has been stable (flat) over the last 5 years after a long period of a declining trend. The trend of *S.* Enteritidis cases in humans ac-quired in the EU has stabilized in 2015–2019. Most of the confirmed salmonellosis cases were acquired in the EU (66.3%), whereas 7.2% reported travel outside EU and 26.5% of infections were of unknown origin. Considering all cases, the highest proportions of domestic cases over 95% were reported by Czechia, Hungary, Latvia, Lithuania, Malta, Portugal, Poland, Slovakia and Spain. The highest proportions of travel-related cases were reported by five Nordic countries: Finland (78.5%), Denmark (64.2%), Sweden (60.9%), Iceland (66.7%) and Norway (76.1%). Among 7900 travel-associated cases with known information on probable country of infection, 80.3% of the cases represented travel outside EU. Turkey, Egypt, Thailand and India were the most frequently reported travel destinations outside EU (15.3%, 10.5%, 10.4% and 6.0%, respectively). In the EU, Spain and Greece were the most common travel destinations. In total, 926 salmonellosis foodborne outbreaks were reported by 23 EU MS in 2019, causing 9169 illnesses, 1915 hospitalizations (50.5% of all outbreak-related hospitalizations) and seven deaths. *Salmonella* caused 17.9% of all food-borne outbreaks during 2019. The vast majority (72.4%) of the salmonellosis foodborne outbreaks were caused by *S.* Enteritidis. The four most implicated food vehicles in strong-evidence salmonellosis foodborne outbreaks were “eggs and egg products’, followed by ‘bakery products”, “pig meat and products thereof” and “mixed food”, as in previous years [1]. In Italy, *S.* Enteritidis is constantly among the main five serovars isolated from humans [2], and it was the seventh most commonly isolated serovar among all animal and food *Salmonella* isolates in Italy in 2018, with an increase over the previous year [3]. Eggs, food containing raw or undercooked eggs, and food of poultry origin in general are usually identified as the confirmed or suspected vehicles responsible both for outbreaks and sporadic cases associated with this serovar. However, Awoke and colleagues reported that *S.* Enteritidis was also isolated from pigs, sheep and rhinoceroses, demonstrating its ability to adapt and infect multiple hosts [4]. Dairy products (including milk and cheese) have been reported as vehicles for human enteric infections, including salmonellosis, but this food category has been implicated in less than 5% of all the *Salmonella* foodborne outbreaks with a known source occurring in the EU from 2010 to 2018, although *S.* Enteritidis was identified in around 40% of them [5].

There is scarce information about the occurrence of *Salmonella* in sheep and the role of raw sheep milk as a source of human salmonellosis. In particular, two outbreaks of *S.* Enteritidis were reported by Pezzotti and colleagues, and in one of them the source was contaminated cheese [6]. On that occasion, the cheese contamination was traced to a farm where two sheep with *S.* Enteritidis mammary infection were identified. Moreover, in 2019 a foodborne outbreak of *Salmonella* Dublin involving thirteen people reporting the consumption of unpasteurized raw sheep milk cheese occurred in France and affected at least one other country, Sweden, with two cases both microbiologically confirmed as connected to the French outbreak using Whole Genome Sequencing [7].

In April-May 2020, an outbreak of *S.* Enteritidis occurred in the Marche region (Central Italy), involving 85 people. The epidemiological investigation carried out by the Prevention Department of the sanitary territorial area 2 of the Marche region identified the raw sheep milk pecorino “primo sale” cheese produced by two local dairies as the most probable source of the outbreak, since all cases declared they had consumed this product.

In this outbreak report, we present the results of the microbiological and environmental investigation conducted in the dairies and the connected sheep farm in order to trace back the source of infection. Moreover, we provide insight into the *S.* Enteritidis infection in sheep and the transmission of this pathogen through milk and raw milk cheese.

## 2. Materials and Methods

### 2.1. Sample Collection and Farm Inspection

Between the beginning of April and the end of May 2020, the Regional Reference Centre for Pathogenic Enterobacteria of the Marche Region (Central Italy) of Istituto Zooprofilattico Sperimentale of Umbria and Marche, department of Tolentino (IZSUM), received 64 human strains of *Salmonella* submitted for serotyping from the regional hospitals’ analysis laboratories of Senigallia, Jesi, Ancona and Civitanova Marche and the private analysis laboratory Ricerche Cliniche of Civitanova Marche, participating in Enter-Net surveillance for Marche Region. The isolates were collected from patients suspected to be involved in an outbreak of salmonellosis. This outbreak involved 85 people, all resident in the Marche Region.

An epidemiological investigation was carried out by the local Prevention Department, and the interviews identified a raw sheep cheese (pecorino “primo sale”) as the potential source of infection.

In the framework of the outbreak investigation, the staff of the Prevention Department collected samples of pecorino “primo sale” cheese at the homes of 10 of the cases, and tracing back of the source of the cheese samples was carried out by the local health authorities.

**Scheme 1:**
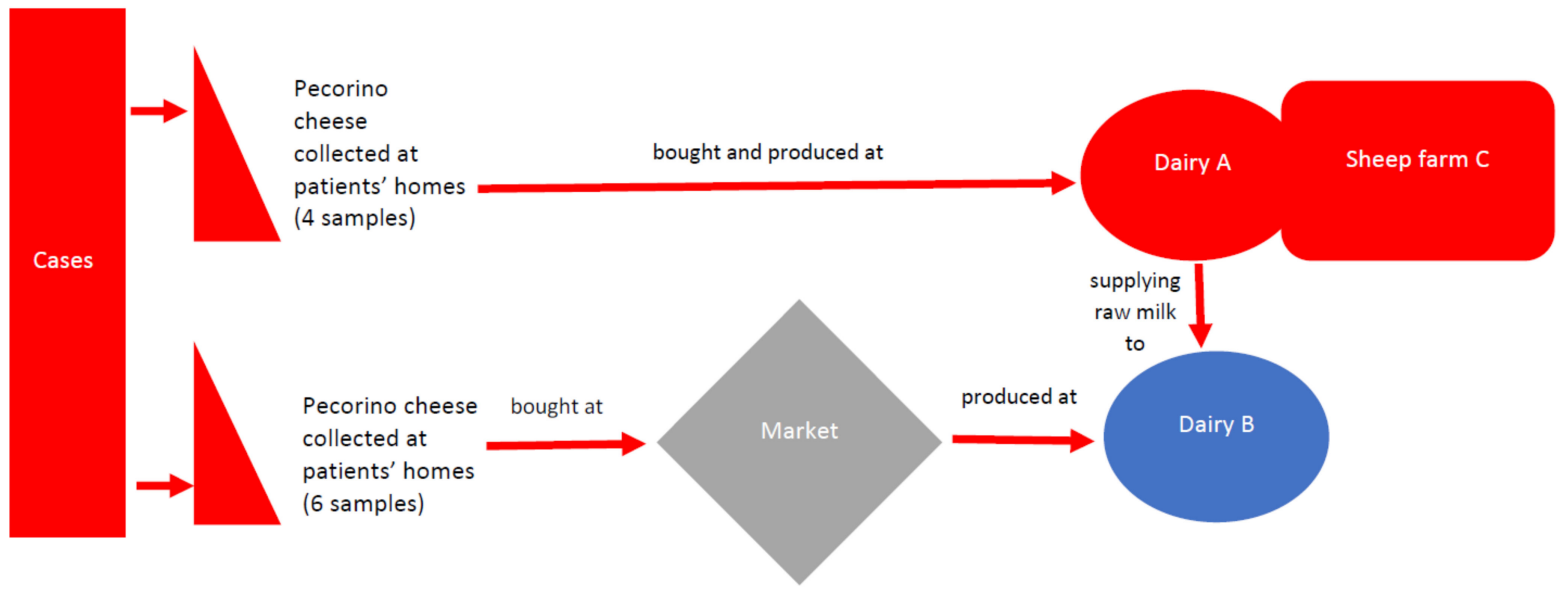
The connections where samples were positive for *S.* Enteritidis are shown in red, the connections where samples were negative are in blue (grey shows the local market where the dairy B cheese was sold but not sampled).

The cheese samples were produced by two different dairies, dairy A (four samples) and dairy B (six samples). Moreover, samples of pecorino “primo sale” cheese belonging to the same batches of the cheese sampled at the cases’ homes were collected from the two dairies. The first one (A) was part of a livestock production facility also including a sheep farm (C) and supplying milk to the second dairy (B). Dairy A sold its cheese directly to the final consumer, while dairy B distributed its products to the local market chain (Scheme 1).

The two dairies (A and B) and farm C (connected to dairy A) were sampled over the period April-October 2020 by the personnel from the Prevention Department, the personnel from the Istituto Zooprofilattico Sperimentale of Umbria and Marche department of Tolentino and the personnel from the private food laboratory analysis Analisi Control of Corridonia (who were responsible for the self-control activities carried out by dairy A and who belong to the Enter-Vet surveillance for Marche Region). The samples included cheese wheels and portions of wheels, milk, sheep faeces and environmental samples (Table 1). All the samples were analysed at the Food Safety Laboratory of IZSUM.

At dairies A and B, whole wheels and portions of wheels of pecorino “primo sale” cheese were collected, for a total of 85 sampling units. At farm C (consisting of a single shed with 239 sheep, open housing in winter and grazing in summer), two sampling visits were performed. During the first visit (23 April) faecal samples from the 239 sheep were collected and pooled into 48 sub-samples (representing 4–5 animals each). Additionally, 4 water samples from the water troughs, 7 environmental samples, 1 bulk milk sample, 1 sample of pigeon faeces and 1 barley feed sample from the silos were collected and analysed (Table 1). On the second visit (19 May), faecal samples were collected from all the 239 sheep and analysed as single samples. Moreover, a total of 24 representative environmental samples including 7 faecal samples taken from the floor, 4 boot swabs and 13 sponge swabs from sheep udders and from different components of the milking equipment present in the breeding facilities were collected. Veterinarians belonging to the local Competent Authority during the first inspection stated that general hygienic standards of the farm were unsatisfactory and the maintenance of milk for more than an hour inside the milking buckets at a temperature of 37 °C before being transferred to the cooling tank appeared to be a dangerous practice. After the sanitization of the farm, on 12 June, 20 faecal samples and 3 boot swabs taken from the stall floor were collected. Twenty-eight samples of bulk milk were collected during different sampling sessions from April to November. Single milk samples from the udder were collected from 40 animals on 7 July and from the remaining 199 animals on 17 July (Table 1).

Single milk samples positive for *Salmonella* spp. were analysed using a most probable number (MPN) technique.

In addition, in November, samples were collected from two *S.* Enteritidis-positive sheep that were culled and brought to the Istituto Zooprofilattico Sperimentale of Umbria and Marche, department of Tolentino: udder milk, supramammary lymph nodes, mesenteric lymph nodes, iliac lymph nodes, intestinal contents, caecal tonsils, nasal content, faeces, bile, mammary gland and mammary secretion (available just for the one sheep that was still lactating) (Table 1).

Stool samples were provided voluntarily by six people who were either owners or employees of dairy A/farm C; none of them showed or declared any symptoms referable to *Salmonella* infection.

### 2.2. Microbiological and Genotypic Analysis

All the samples collected at the dairies and at the farm as well as cheese samples from the homes of the people involved were analysed for *Salmonella* detection according to UNI EN ISO 6579-1:2017.

All the samples taken from the two sheep subjected to anatomical-pathological examination were analysed for *Salmonella* detection according to an internally validated method.

All *Salmonella* isolates from both veterinary-food and human samples were serotyped according to ISO/TR 6579-3:2014.

The MPN (most probable number) analysis of the single milk samples positive for *Salmonella* were performed according to ISO 7218 and to UNI EN ISO 6887 all parts.

MLVA analyses of all the *S.* Enteritidis isolates were carried out by the National Reference Centre for Salmonellosis of Istituto Zooprofilattico Sperimentale delle Venezie and Department of Infectious Diseases of Istituto Superiore di Sanità for food, animal and environmental samples and human samples, respectively following the Laboratory SOP by ECDC [8]. MLVA profiles were reported as a string of 5 characters (SENTR7-SENTR5-SENTR6-SENTR4-SE-3) representing the number of repeats at the corresponding locus.

### 2.3. Antimicrobial Susceptibility

Antimicrobial susceptibility for the 64 human origin *S.* Enteritidis isolates and the 59 food, animal or environmental origin *S.* Enteritidis isolates was determined by the disk diffusion method, according to the Clinical and Laboratory Standards Institute guidelines (CLSI, 2020) using ampicillin (AMP 10 µg), amoxicillin + clavulanic acid (AMC 30 µg), cefotaxime (CTX 30 µg), ceftazidime (CAZ 30 µg), cefoxitin (FOX 30 µg), chloramphenicol (C 30 µg), gentamicin (CN 10 µg), meropenem (MEM 10 µg), tetracycline (TE 30 µg), trimethoprim + sulfamethoxazole (SXT 23.75/1.25 µg), trimethoprim (TMP 5 µg), sulfisoxazole (ST 300 µg), pefloxacin (PEF 5 µg) and streptomycin (S 10 µg). The reference strain *Escherichia coli* ATCC 25922 for antimicrobial susceptibility was used.

The CLSI interpretive criteria for disk diffusion susceptibility testing of *Salmonella* (CLSI, 2020) were used [9].

## 3. Results

**Figure 1:**
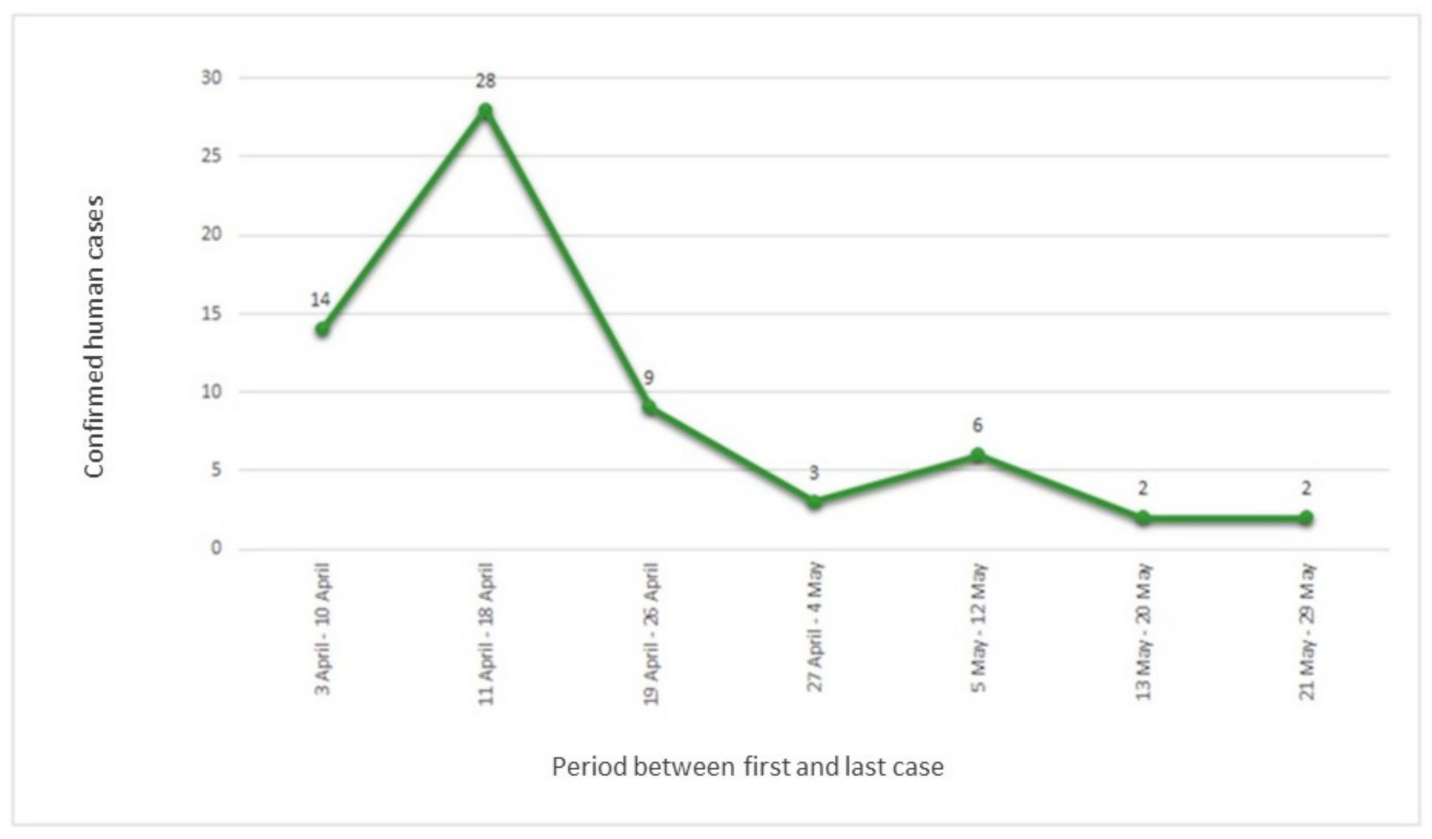
Epidemic curve of *S.* Enteritidis confirmed human cases in function of the time period.

In April–May 2020, an outbreak of *S.* Enteritidis occurred in the Marche region (Central Italy) involving 85 people, 64 of whom were microbiologically confirmed. Figure 1 shows the epidemic curve of the confirmed *S.* Enteritidis cases; an exponential trend with a peak in April 2020 (54 confirmed cases) and, subsequently, a decrease in May was observed.

The 64 human *Salmonella* strains received by IZSUM were serotyped as *S.* Enteritidis.

All ten samples of pecorino “primo sale” cheese collected from the cases’ homes and 18 of the 25 pecorino “primo sale” cheese samples collected at dairy A were positive for *S.* Enteritidis, while all 60 samples collected at dairy B were negative for *S.* Enteritidis (Table 1).

One of 48 faecal pools and the bulk milk, sampled on 23 April, were positive for *S.* Enteritidis. All water samples from the water trough, the sample of barley, sample of pigeon faeces and all the environmental samples were negative (Table 1).

On 19 May, all the faeces analysed as a single sample were negative, while 3 of the 7 faecal samples taken from the floor together with 2 of the 4 boot swabs were positive for *S.* Enteritidis. All sponge swabs (13) made from pools of different sheep udders and from different components of the milking equipment were negative (Table 1).

Until July, all samples of bulk milk collected before and after the environmental sanitization procedures (27) were positive for *S.* Enteritidis.

Two of 239 samples of udder milk, collected during the two sampling sessions on 7 and 17 July, were positive for *S.* Enteritidis. These two samples were also analysed quantitatively using a MPN technique, and numbers on 7 and 17 July were estimated as 43 MPN/mL and 0.36 MPN/mL, respectively. The isolation of *S.* Enteritidis from udder milk led to the culling of these two sheep. Bulk milk sampled after their removal was negative for *S.* Enteritidis (Table 1). Only one mammary secretion sample collected from one of the two culled sheep was positive for *S.* Enteritidis; all the other samples collected from the carcasses were negative (Table 1).

All the *S.* Enteritidis isolates of human, animal, environmental or food origin (129 in total) were pan-susceptible to all tested antimicrobials.

All stool samples (6) provided by the owners and employees of dairy A/farm C were negative for *Salmonella* spp. (Table 1).

All the *S.* Enteritidis isolates of human, animal, environmental or food origin (19 in total) showed the same MLVA profile corresponding to 2-9-10-4-2.

## 4. Discussion

Our study provided evidence of intestinal and udder infection due to *S.* Enteritidis in sheep with consequent shedding of *S.* Enteritidis with milk. Moreover, contaminated raw sheep milk used to made pecorino “primo sale” cheese was the source of a foodborne outbreak involving 85 people.

In recent years, and with changing habits, consumers increasingly tend to prefer foods that are not processed or, even better, that are not subjected to heat treatments, as such foods are considered to have enhanced nutritional qualities, taste and health benefits. This phenomenon has also affected the dairy sector, leading it to increase the offer of raw milk and raw milk products, often using traditional production methods. Meaningful differences in nutritional values between pasteurized and unpasteurized milk have not demonstrated, and other purported benefits of raw milk consumption have not been substantiated. Conversely, the role of unpasteurized dairy products in the transmission of infectious diseases has been established repeatedly. Disease outbreaks associated with consumption of raw milk are considerably more frequent than outbreaks associated with the consumption of pasteurized milk [10].

In 2015, EFSA published a scientific opinion on public health risks associated with raw milk, concluding that raw milk can be a source of harmful bacteria, mainly *Campylobacter*, *Salmonella*, Shiga toxin producing *Escherichia coli* (STEC) and *Brucella melitensis*. Data reported to EFSA (2011–2015) show an overall prevalence of *Salmonella* spp. in any type of milk (raw, pasteurized, cow, goat, sheep) equal to 0.1%. However, data on the prevalence of *Salmonella* spp. in sheep milk are scarce [11]. The meta-analysis study carried out by Gonzales-Barron et al. (2017) estimated an overall prevalence of *Salmonella* spp. in raw sheep milk equal to 1.4% (95% confidence interval (CI:0.3–6.6%) based on the results of four studies [12]. Moreover, data collected by EFSA showed a prevalence of 0.2% for *Salmonella* spp. in cheese in general, as well as in cheeses made from sheep milk, and raw sheep milk was identified as a main source of *Salmonella* spp., as was the case for products made thereof [11].

Contaminated dairy products such as milk and cheese have been demonstrated as sources of *S.* Enteritidis to consumers [13], as reported by Pezzotti and colleagues [6] and as described during a recent outbreak reported in France [7]. Hence, *Salmonella* infection in sheep is a real risk for public health considering that sheep milk is generally not pasteurized before cheese production and that *S.* Enteritidis can persist in the products for several months [6]. The microbiological and environmental investigation together with the molecular typing of *S.* Enteritidis isolates, carried out in the framework of the described outbreak, confirmed the raw sheep milk cheese as the source of infection.

Once food personnel were ruled out as healthy carriers after they tested negative for *Salmonella*, our primary hypothesis was that intestinal colonization of some sheep could have resulted in faecal shedding and subsequent dissemination of *S.* Enteritidis in the farm and dairy environment. Following on, most sheep could be negative for *Salmonella*, but some sheep could prove to be carriers and intermittent shedders. Shedding of the pathogen at extremely low levels resulted in the low frequency of positive faecal samples collected from the individual animals, which hampered the identification of the *Salmonella*-positive sheep. Moreover, the environmental faecal contamination associated with poor management at farm and dairy levels could have been the cause of milk contamination.

However, the persistent isolation of *S.* Enteritidis in milk samples after the farm sanitization procedures led us to formulate a second hypothesis of mammary gland contamination that could result in shedding of *S.* Enteritidis with the milk.

According to the results of farm inspection, official veterinarians verified that the producer did not immediately transfer the milk to the production process, but the milk was held for more than an hour inside the milking buckets before being transferred to the cooling tank. Therefore, the milk remained at 37 °C (exit temperature from the udder), which would provide optimal conditions for *Salmonella* recovery and proliferation, likely leading to a sharp increase of the contamination level.

The finding that 2 of 239 udder milk samples contained *S.* Enteritidis confirmed the hypothesis that after the intestinal colonization, the pathogen located in the mammary gland and was shed with the milk.

In addition, one of two udder milk samples from the two *S.* Enteritidis-positive sheep (that were later culled) tested negative after these sheep were removed from the milking flock. This may be explained by the low bacterial load in this negative sample, also related to the small quantity of milk available at the time of collection (the sheep was dry), or by the self-resolution of the udder infection after the sheep was removed. Both these hypotheses could also explain the reason why, after the culling, all the samples collected from this sheep carcass tested negative for *Salmonella*. On the contrary, it is anomalous that, regarding the lactating sheep, the mammary gland and the mammary lymph nodes tested negative for *Salmonella* in the face of a positive mammary secretion.

The first considerations made were about the repeated positivity of bulk milk and cheese samples, the absence of udder contamination, the great difficulty in isolating *Salmonella* from faeces taken from single sheep and, on the contrary, the repeated positivity of faeces taken from the floor and of boot swabs at farm level. Regarding the negativity of all the cheese samples (60) collected at dairy B, this finding was related to the subsequent assessment that the sampled cheeses were produced with milk obtained from suppliers other than dairy A.

Furthermore, the *S.* Enteritidis positivity of milk, both milked and collected from the udder after culling, together with the maintenance of milk for more than an hour inside the milking buckets at a temperature of 37 °C before it was transferred to the cooling tank, would explain the high levels of *S.* Enteritidis contamination in the bulk milk. The use of the raw, unpasteurized contaminated bulk milk for cheese production then resulted in the pathogen’s presence in the “primo sale” pecorino cheese.

Although the hygienic standards of the farm were unsatisfactory according to the first on-farm inspections, samples of animal feed, water and barley tested negative for *Salmonella*; the pigeon faeces present in the breeding locales were also negative. Therefore, the original source of the *S.* Enteritidis remains unknown.

On the contrary, the adequate cleaning of milking equipment, shown by the negativity of the sponge swabs from different components of the milking equipment, explains the mammary colonization as a consequence of *S.* Enteritidis migration from the intestine to the mammary gland.

The identification of a unique MLVA profile of the investigated *S.* Enteritidis isolates confirmed the epidemiological relationship among human and animal cases and the cheese as the source of human infection. This MLVA profile (2-9-10-4-2) has been never observed before in the Italian human database, Enter-Net, or in the food-veterinary database, Enter-Vet. Furthermore, our analysis of the ECDC database of the MLVA profiles indicates that 2-9-10-4-2 was detected in only one case in 2018, with no linked cases.

## 5. Conclusions

Satisfactory hygienic standards and the adoption of prevention measures and good processing techniques in farms and dairies are fundamental for preventing sheep milk contamination with *Salmonella*, especially if raw milk is destined for human consumption or is used in further production that does not include a pasteurization step.

First, good hygienic standards during milking are required, with attention to the cleaning of the personnel’s hands and of animals’ udders, the correct cleaning and disinfection of milking equipment and raw milk storage equipment, and the scrupulous washing and effective disinfection of the premises and tools used for processing.

At the same time, it is extremely important to cool raw milk immediately, to keep hot milk strictly separate from chilled milk and to securely maintain the raw milk cold chain until the milk is released from the cold chain for processing.

In any similar outbreak, we recommend sampling the milk from single animals as soon as possible in order to identify positive animals and to put in place the correct preventive measures.

## Figures and Tables

**Table 1:** Number of *S.* Enteritidis-positive samples out of the total of animal, environmental and food samples collected.

Date of Sampling (Year 2020)	N° of Samples	N° of *S.* Enteritidis-Positive Samples
13 April	1 bulk milk	1
15–21 April	4 samples of pecorino “primo sale” cheese (dairy A) collected at homes of patients	4
15–29 April	6 samples of pecorino “primo sale” cheese (dairy B) collected at homes of patients	6
23 April	25 cheese wheels from dairy A	18
	60 cheese portions from dairy B	0
	48 pooled faecal samples from 239 sheep	1
	4 water samples from the water troughs	0
	7 environmental samples	0
	1 barley feed sample from silos	0
	1 bulk milk	1
	1 sample of pigeon faeces	0
24 April	1 bulk milk	1
27 April	1 bulk milk	1
19 May	7 faecal samples taken from the floor	3
	4 boot swabs	2
	13 sponge swabs from sheep udders and milking equipment	0
	239 faecal samples from individual sheep (single samples analysis)	0
From May to 12 June	17 samples of bulk milk	17
12 June Sanitization procedures		
From 13 June to 27 July	6 samples of bulk milk	6
13 June	20 faecal samples taken from the floor	0
13 June	3 boot swabs	0
7 July	40 individual samples of udder milk	0
17 July	199 individual samples of udder milk	2
7 August	1 bulk milk after removal of two *S.* Enteritidis-positive sheep	0
13 October 2020	2 samples of udder milk from the two *S.* Enteritidis-positive sheep	1
26 November 2020	Samples from the two culled *S.* Enteritidis-positive sheep: from supramammary lymph nodes, mesenteric lymph nodes, iliac lymph nodes, intestinal contents, caecal tonsils, nasal content, faeces, bile, mammary gland, mammary secretion (available just from the one sheep that was still lactating)	1 (mammary secretion of the lactating sheep)
